# Associations between meteorological factors and COVID-19: a global scoping review

**DOI:** 10.3389/fpubh.2024.1183706

**Published:** 2024-07-18

**Authors:** Jesse Limaheluw, Sophia Dollmann, Sofia Folpmers, Lola Beltrán Beut, Afroditi Lazarakou, Lucie C. Vermeulen, Ana Maria de Roda Husman

**Affiliations:** ^1^Centre for Infectious Disease Control, National Institute for Public Health and the Environment (RIVM), Bilthoven, The Netherlands; ^2^Institute for Risk Assessment Sciences (IRAS), Utrecht University, Utrecht, The Netherlands

**Keywords:** SARS-CoV-2, respiratory diseases, airborne viruses, climate, weather, pandemic preparedness

## Abstract

**Background:**

Many respiratory viruses and their associated diseases are sensitive to meteorological factors. For SARS-CoV-2 and COVID-19, evidence on this sensitivity is inconsistent. Understanding the influence of meteorological factors on SARS-CoV-2 transmission and COVID-19 epidemiology can help to improve pandemic preparedness.

**Objectives:**

This review aimed to examine the recent evidence about the relation between meteorological factors and SARS-CoV-2/COVID-19.

**Methods:**

We conducted a global scoping review of peer-reviewed studies published from January 2020 up to January 2023 about the associations between temperature, solar radiation, precipitation, humidity, wind speed, and atmospheric pressure and SARS-CoV-2/COVID-19.

**Results:**

From 9,156 initial records, we included 474 relevant studies. Experimental studies on SARS-CoV-2 provided consistent evidence that higher temperatures and solar radiation negatively affect virus viability. Studies on COVID-19 (epidemiology) were mostly observational and provided less consistent evidence. Several studies considered interactions between meteorological factors or other variables such as demographics or air pollution. None of the publications included all determinants holistically.

**Discussion:**

The association between short-term meteorological factors and SARS-CoV-2/COVID-19 dynamics is complex. Interactions between environmental and social components need further consideration. A more integrated research approach can provide valuable insights to predict the dynamics of respiratory viruses with pandemic potential.

## 1 Introduction

Respiratory viruses such as influenza are known to be sensitive to meteorological factors (1, 2). Through direct effects on virus viability, and indirect (behavioral) effects such as indoor crowding during bad weather, factors like ambient temperature also influence the epidemiology of their associated diseases (3). The influence of meteorological factors is likely to play a role in shaping the observed seasonality of these diseases in temperate climates (4, 5).

Several studies have pointed toward climate sensitivity of diseases caused by human coronaviruses (HCoV) as well. Peaks in the incidence of severe acute respiratory syndrome (SARS) were observed during periods of colder weather (6), while incidence of Middle East respiratory syndrome (MERS) was observed to increase during warmer and drier conditions (7). Sunshine hours and air temperatures were found to be negatively associated with disease caused by HCoV-HKU1, HCoV-NL63, HCoV-OC43 and HCoV-229E (8).

Since the beginning of the Coronavirus disease 2019 (COVID-19) pandemic, it has been hypothesized that COVID-19 could become a seasonal disease such as influenza (9). Experimental results have indeed shown that Severe Acute Respiratory Syndrome Coronavirus-2 (SARS-CoV-2) is sensitive to meteorological factors, such as sunlight (10). However, large discrepancies exist between epidemiological study results (11, 12).

It thus remains unclear how meteorological factors influence SARS-CoV-2 transmission, and epidemiology of COVID-19. However, understanding this influence is important to improve epidemiological predictability, both in the short and long term (e.g., in the context of climate change). For other respiratory viruses with pandemic potential such insights might be useful for pandemic preparedness and response. Here, we present a scoping review to further examine what is known about the relation between meteorological factors and COVID-19 (transmission and outcomes/epidemiology) including (in)direct effects on SARS-CoV-2. Direct effects are impacts on the SARS-CoV-2 pathogen itself (e.g., virus viability), while indirect effects relate to factors accountable for SARS-CoV-2 spread (e.g., distance over which droplets are dispersed). This scoping review aims to provide an overview of the existing knowledge on the relationship between meteorological factors and SARS-CoV-2/COVID-19 and identify the main knowledge gaps in this research area.

## 2 Methods

### 2.1 Search strategy and selection criteria

A review protocol was developed following the Preferred Reporting Items For Systematic Reviews And Meta-analyses (PRISMA) guidelines, specifically the extensions for Systematic Review Protocols (PRISMA-P) and for Scoping Reviews (PRISMA-ScR) (13, 14). Literature was first collected on January 6th, 2021, and again on May 12, 2023 to include studies published up to January 2023. We used the scientific literature databases Embase, PubMed, and Scopus. A broad search query consisting of two main concepts was used: (I) short-term meteorological factors and (II) SARS-CoV-2. The search query considered multiple human coronaviruses because we expected more findings on the relation with meteorological factors. However, these were ultimately excluded from the study due to the comparatively small number of publications. Where possible, index terms (Emtree for Embase, or MESH for Pubmed) were included (Section 1 of Supplementary Data Sheet 1).

Peer-reviewed studies published in English between January 2000 and January 2023 were considered in this review. Only full research articles were accepted. Eligibility criteria were identical during two screening phases: a publication was included if it studied the effects of one or multiple meteorological factors on SARS-CoV-2 or COVID-19. A publication was excluded if it did not mention a relation to a meteorological factor, if it only studied an effect of long-term climate change, or if it did not present any quantitative outcomes. Preprints were not considered.

### 2.2 Screening and data extraction

Following the collection of literature, duplicate records were removed. Both titles and abstracts, and all full texts of retained publications were screened twice by two independent reviewers. Disagreements were resolved through discussion between the two reviewers. Data extraction was performed during full text screening using a data extraction form (Table 1). Non-meteorological variables that were measured in relation to human coronaviruses were collected to account for potential confounding factors and effect modifiers. For literature collected in the first search, Endnote version 20 was used to manage references and to screen titles and abstracts. Considering the large number of additional records to screen following the second search, the AI-tool ASReview (15) was used for the second round of title and abstract screening. This tool, described in detail in van de Schoot et al. (15), uses machine learning algorithms to accelerate title and abstract screening based on manual input by the reviewers. After having screened at least 20% of all records (15, 16), we stopped screening, following consecutive exclusion of 100 papers. Google Docs was used for data extraction.

**Table 1:** Structure of the data-extraction form.

Main category	Information extracted from main category
General descriptive field	Paper title; Study Region; Country; Study type (observational or experimental/simulated); Time period
Methods	Outcome measures (cases, deaths, hospitalizations, other); Significance reported; Interactions; Non-meteorological variables
Results	Associations of meteorological factors; Explanation of associations per meteorological factor; Notes

Individual associations were extracted from each included publication. For example, if a publication contained specific results for three locations and two outcome measures, six individual associations were collected. Extraction focused on correlation coefficients, but other types of associations were included if no correlation tests were conducted. Associations were classified as positive, negative, non-significant, or variable, considering the impact on COVID-19. A positive association thus describes or implies that an increase in a meteorological variable would lead to an increase in COVID-19. We distinguished between (primarily observational) studies about COVID-19 (cases, deaths, hospitalizations etc., excluding recovery outcomes), and experimental and simulation studies that assessed effects on SARS-CoV-2. For observational studies, each association was assigned to a World Health Organization (WHO) region and if possible, to a country and/or a specific location within a country. We extracted specific location or country centroid coordinates using a geoparser. For associations that could be assigned to a specific location, we also determined the Köppen-Geiger climate zone of that location. For this, we only considered the main climate zones: tropical, arid, temperate, continental, and polar (17, 18). For each meteorological variable, we calculated an average direction of association per WHO region and per climate zone, as well as for specific locations for which more than one association was identified. To do this, we assigned a score of one to positive associations, a score of negative one to negative associations, and a score of zero to non-significant associations. Variable associations were excluded from this specific analysis. Some studies provided single associations for multiple regions or the entire world. These were included in the overall results, but not considered in the regional analyses. We did not consider study type or statistical methodology when grouping studies.

## 3 Results

**Figure 1:**
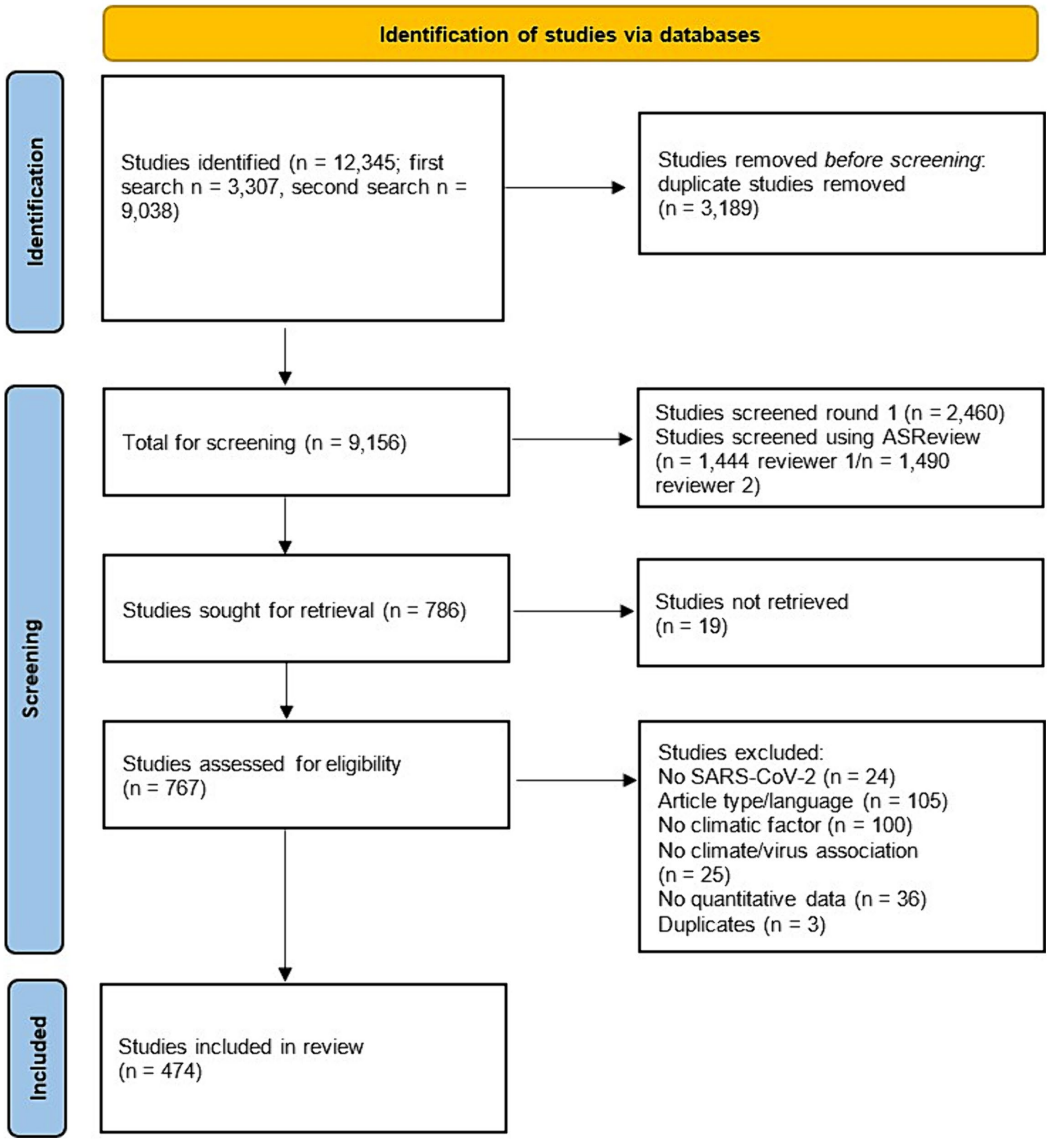
PRISMA flow diagram of study selection process.

A total of 9,156 studies remained after deduplication. Titles and abstracts of all 2,460 studies from the initial search were screened. Using ASReview ~22% (1,490 articles for reviewer 1 and 1,444 for reviewer 2) of the articles collected during the second search were screened. Following full text screening 474 articles on SARS-CoV-2 or COVID-19 were included in the scoping review (Figure 1). The full data extraction outcomes are presented in Supplementary Table S1.

### 3.1 Characteristics of included studies

**Figure 2:**
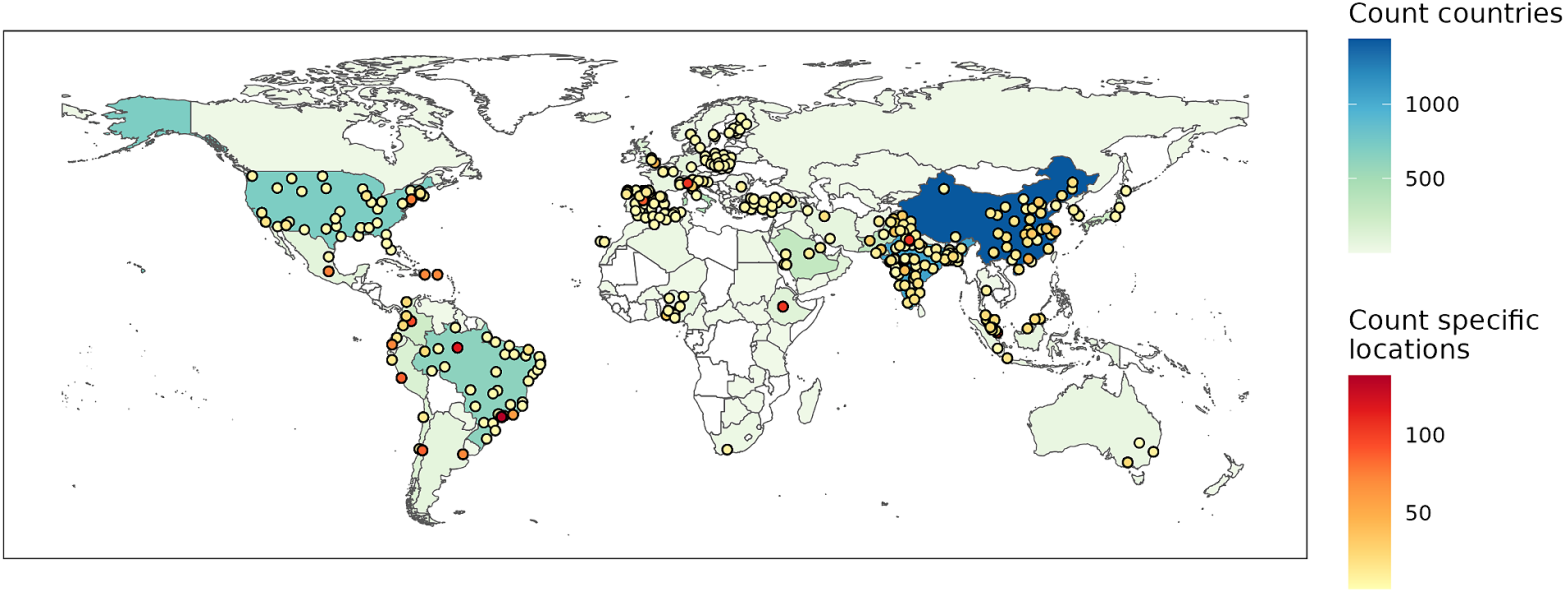
Number of associations found for specific geographic locations (points) or countries.

Most included studies used an observational time series study design (445/474). With one exception, these studies examined COVID-19. Twenty-nine articles presented experimental or simulated results. These studies all pertained to SARS-CoV-2. A total of 10,643 individual associations were extracted. In the non-experimental studies, we found results for 103 countries and 393 specific locations (Figure 2). China, India, and Brazil were the best represented countries, while São Paolo (Brazil), Manaus (Brazil), and Delhi (India) were the specific locations for which most associations were found. Over 1,000 associations were identified for all WHO regions apart from the African region.

**Figure 3:**
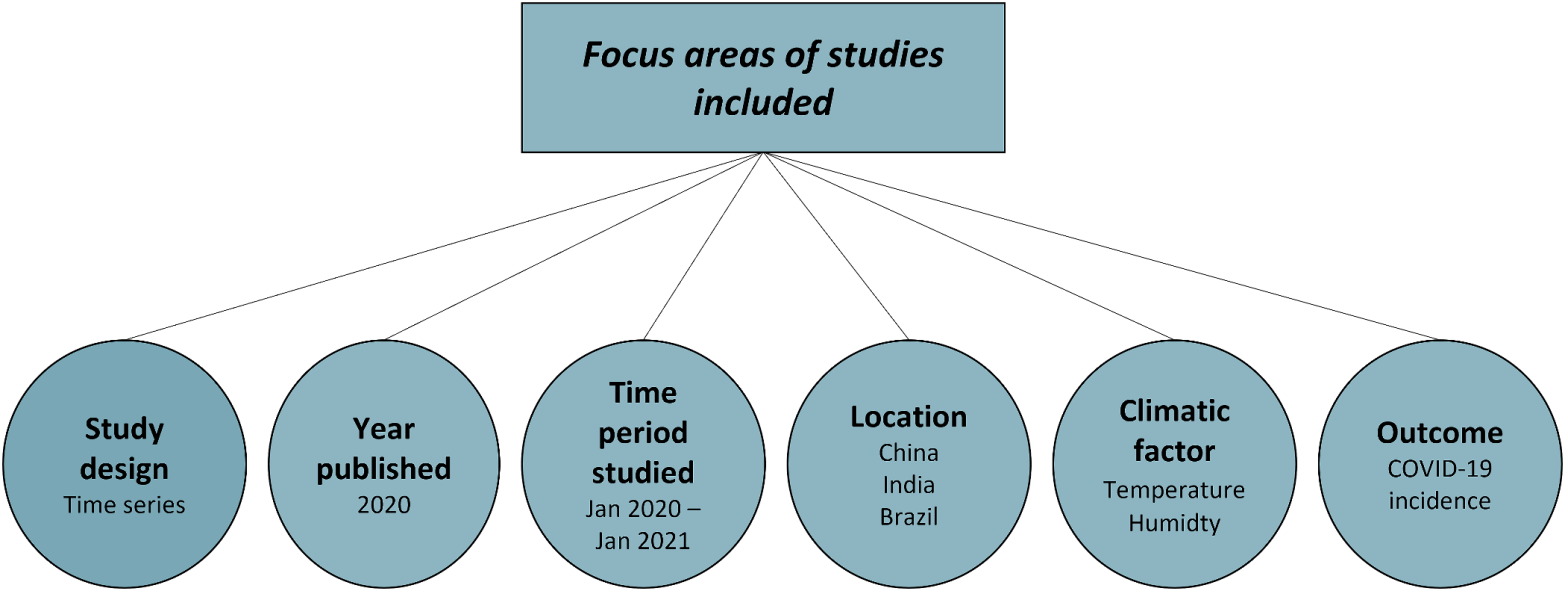
Overview of the overall focus of the studies included.

Figure 3 represents the overall focus areas of the articles included in the scoping review. The evidence was primarily observational in nature and focused on 2020, the first year of the COVID-19 pandemic. Associations with temperature and humidity were studied most often.

### 3.2 SARS-CoV-2/COVID-19 climate sensitivity

#### 3.2.1 Evidence from experimental and simulation studies on SARS-CoV-2

In total, 29 studies provided evidence from experimental and simulation studies on SARS-CoV-2 (Table 2). Twenty-four experimental studies evaluated the direct effects (i.e., inactivation) of temperature, humidity, UV radiation, and wind in a controlled environment on SARS-CoV-2. Some studies examining multiple factors simultaneously point toward interaction between variables. Experimental studies assessed effects on SARS-CoV-2 in different matrices, such as air, biological fluids, surfaces, and objects (e.g., bank notes).

**Table 2:** Associations between meteorological factors and SARS-CoV-2.

Meteorological factor	Reference	Experimental setting	Association
Experimental studies			
Temperature	(19)	Stability of SARS-CoV-2 on a plastic surface	Negative
Temperature	(20)	Persistence of SARS-CoV-2 on common surfaces, such as polymer bank notes, paper banknotes, brushed stainless steel, glass, vinyl and cotton cloth	Negative
Temperature	(21)	Stability of SARS-CoV-2 on a plastic surface	Negative
Temperature	(22)	Stability of virus like particles on a functionalized glass surface	Negative
Temperature	(23)	Inactivation of SARS CoV-2 on porous and non-porous materials (hard- and softback book covers, plain paper pages, plastic protective cover and DVD case)	Negative
Temperature	(24)	Stability of SARS-CoV-2 Alpha and Delta variants on stainless steel	Negative
Temperature	(25)	Affinity of SARS-CoV-2 Spike glycoprotein using biochemical, biophysical, and functional assays	Negative
Temperature	(26)	SARS-CoV-2 transmission between naive golden Syrian hamsters and infected hamsters under controlled environmental conditions	Positive
Temperature	(27)	SARS-CoV-2 stability in spiked human nasal mucus, sputum, saliva, tears, blood, semen and human urine from healthy donors	Negative
Temperature	(28)	SARS-CoV-2 isolates in aerosols were compared in a rotating drum chamber	Negative
Temperature	(29)	Cellular infectivity experiment using SARS-CoV-2 on cotton gauze, dried, and then incubated under specific temperature conditions	Negative
Temperature	(30)	Droplet evaporation experiments in an environmental chamber	No significant effect
Temperature	(27)	SARS-CoV-2 stability on cloth, concrete, polypropylene, stainless steel, galvanized steel, nitrile gloves, Tyvek, N95 mask, Styrofoam, cardboard, rubber, and glass	Negative
Temperature	(31)	Persistence of SARS-CoV-2 on stainless steel	Negative
Temperature / Humidity	(32)	SARS-CoV-2 in a simulated clinically relevant matrix dried on nonporous surfaces	Negative
Temperature / Humidity	(33)	Droplets/aerosols with varying diameters and rates of size reduction of SARS-CoV-2 spike pseudovirus-laden droplets/aerosols size were explored in a 1.5 m × 1.0 m × 1.2 m laboratory exposure chamber	Negative
Temperature / Solar radiation / Humidity	(34)	SARS-CoV-2 in aerosols across a range of temperature, humidity, and simulated sunlight levels using an environmentally controlled rotating drum aerosol chamber	Negative
Solar radiation	(10)	Aerosolized SARS-CoV-2 under simulated sunlight	Negative
Solar radiation	(35)	Model estimation of SARS-CoV-2 inactivation by artificial UVC and by solar ultraviolet radiation	Negative
Solar radiation	(36)	Viability of SARS-CoV-2 in mucus and deposited on stainless steel	Negative
Solar radiation	(37)	Fresh samples of a nasopharyngeal swab positive (ascertained by PCR) for SARS-CoV-2 have been exposed to sunlight	No (significant) effect
Solar radiation	(28)	SARS-CoV-2 stability in aerosols in a rotating drum chamber	Negative
Solar radiation	(31)	Persistence of SARS-CoV-2 on stainless steel	Negative
Humidity	(10)	Aerosolized SARS-CoV-2 under simulated sunlight	No significant effect
Humidity	(21)	SARS-CoV-2 inactivation on a polypropylene plastic surface	Variable
Humidity	(26)	Naive golden Syrian hamsters are placed on the opposite side of a porous, double-walled, barrier from infected hamsters for a defined duration under controlled environmental conditions	Positive
Humidity	(28)	SARS-CoV-2 isolates in aerosols were compared in a rotating drum chamber	Positive
Humidity	(38)	SARS-CoV-2 aerosols exposed to controlled humidity	Positive
Humidity	(30)	Droplet evaporation experiments in an environmental chamber	Negative
Simulation studies			
Wind	(39)	Droplet evaporation model	Positive
Temperature / Humidity / Wind	(40)	Fluid dynamics simulations to calculate the concentration rate of SARS-CoV-2 particles in contaminated saliva droplets	Negative
Temperature / Humidity	(41)	Computational fluid dynamics models to predict airborne exposure to the SARS-CoV-2 virus from a coughing person in a mechanically ventilated room	Variable
Humidity	(42)	Indoor air mass-balance model (modified Wells-Riley model) to include the impact of RH on the volume emission of respiratory droplets from an infected individual and its removal mechanisms of deposition by gravitational settling and inactivation by biological decay	Variable
Humidity	(43)	Modified Wells-Riley model to simulated the size distribution and dynamics of SARS-CoV-2 emitted from a speaking person in a typical residential setting	Variable

The majority (15/17) of studies investigating the effects of temperature on SARS-CoV-2 found a negative effect, independent of the virus variant. This indicates that higher temperatures increase the rate of virus inactivation. Fourteen studies assessed only the effect of temperature, whereas the remaining three studies examined the effects (and interactions) of temperature and other meteorological factors simultaneously. One of the studies investigating only temperature looked at the persistence of SARS-CoV-2 on a plastic surface and found a faster decay of the virus at higher temperatures (19). Riddell and colleagues also explored the persistence of SARS-CoV-2 on surfaces and confirmed the higher rate of SARS-CoV-2 inactivation with increasing temperature (20). They showed that SARS-CoV-2 could remain infectious on surfaces at 20°C for up to 28 days, but only survived for 5–10.5 h (depending on the surface material) at higher temperatures (40°C). Additionally, Biryukov and colleagues showed negative associations of temperature and relative humidity (RH) on SARS-CoV-2 on surfaces, such as stainless steel or plastic (24 to 35°C, 20 to 60% RH) (32). Dabisch and colleagues demonstrated that temperature, humidity, and sunlight significantly affected the viability of aerosolized SARS-CoV-2, supporting that higher temperature and solar radiation are associated with higher inactivation rates (34). They demonstrated that the magnitude of the effect of humidity on the infectivity of SARS-CoV-2 aerosols is amplified when temperature or sunlight are increased. Only one study presented a positive association between temperature and SARS-CoV-2 (26). This was the only experimental study which investigated the effects on the transmission of SARS-CoV-2 (between hamsters). One study did not find a significant effect (30). Except for one study without significant results (37), all experiments assessing the influence of solar radiation on SARS-CoV-2 showed negative effects on virus viability. Schuit and colleagues studied SARS-CoV-2 in aerosols and demonstrated increased inactivation due to simulated sunlight, but no effect of humidity (10). Sagripanti and Lytle found comparable results on viral inactivation due to higher solar radiation (35).

In comparison to temperature and solar radiation, which showed clear effects on virus inactivation, humidity is the only meteorological variable with divergent findings. However, the majority (3/6) showed a positive association between humidity and SARS-CoV-2. One study found a negative association between indoor humidity and droplet evaporation (30). Another study examining the effect of humidity on SARS-CoV-2 inactivation showed variable effects, with the highest estimated virus half-life at 40% RH and 85% RH (21).

Five simulation studies modeled the dynamics of virus-containing droplets. Regarding the association between wind and SARS-CoV-2, Li and colleagues who studied the dispersion of cough droplets in a tropical environment, demonstrated that smaller droplets can be transported over longer distances at higher wind speed. They observed a higher virus deposition on a person, which can enable secondary transmission (39). Dbouk and colleagues (40) who examined the combined effect of wind speed, temperature, and relative humidity on the virus concentration rate detected a negative effect. Another fluid dynamics model modeling the airborne exposure to the SARS-CoV-2 virus from a coughing person yielded variable results (41). Increasing the relative humidity in a mechanically ventilated room resulted in a statistically significant reduction in the exposure. At lower temperature and higher relative humidity (16°C and 70% RH), droplets generally traveled the shortest distance as they deposited faster to the floor. Although a more rapid evaporation was observed at a higher temperature (RH = 30 and 50%), the effect of RH on evaporation was more important than the effect of temperature.

Two studies simulating the effects of humidity on the airborne transmission of SARS-CoV-2 viral load showed non-monotonic effects. In both studies, the risk of infection was highest at 37% RH (42, 43).

#### 3.2.2 Evidence from observational studies on COVID-19

**Figure 4:**
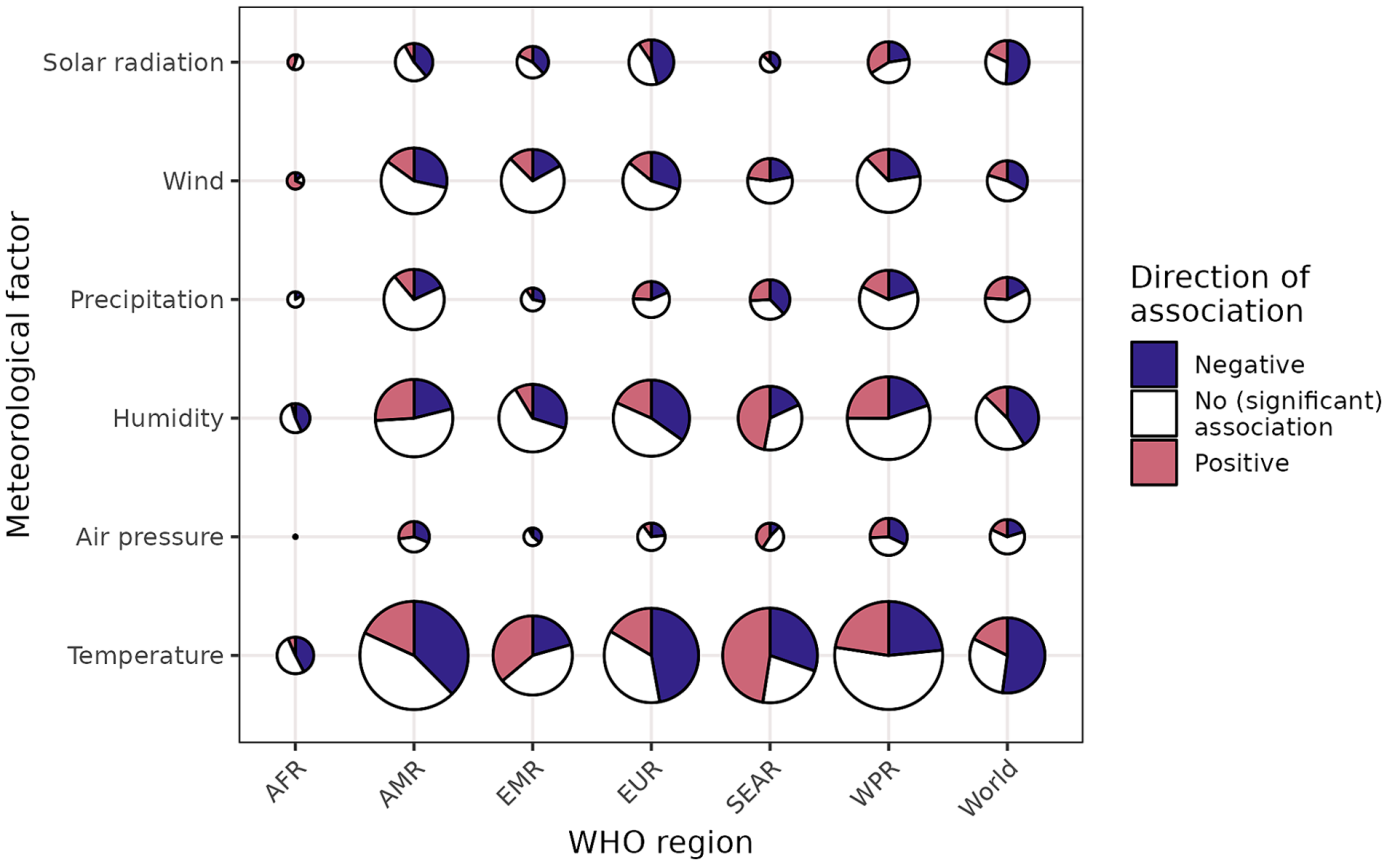
Bubble plot of the associations between meteorological factors and COVID-19 per WHO region. Bubble size represents the number of associations. The maximum number of associations found for a specific region-factor combination was 934 (temperature for the Americas region), and the smallest number greater than 0 was 1 (e.g., air pressure for the African region). AFR, African region; AMR, Americas region; EMR, Eastern Mediterranean region; EUR, European region; SEAR, South-East Asia region; WPR, Western Pacific region; World, associations pertaining to multiple or all regions.

**Figure 5:**
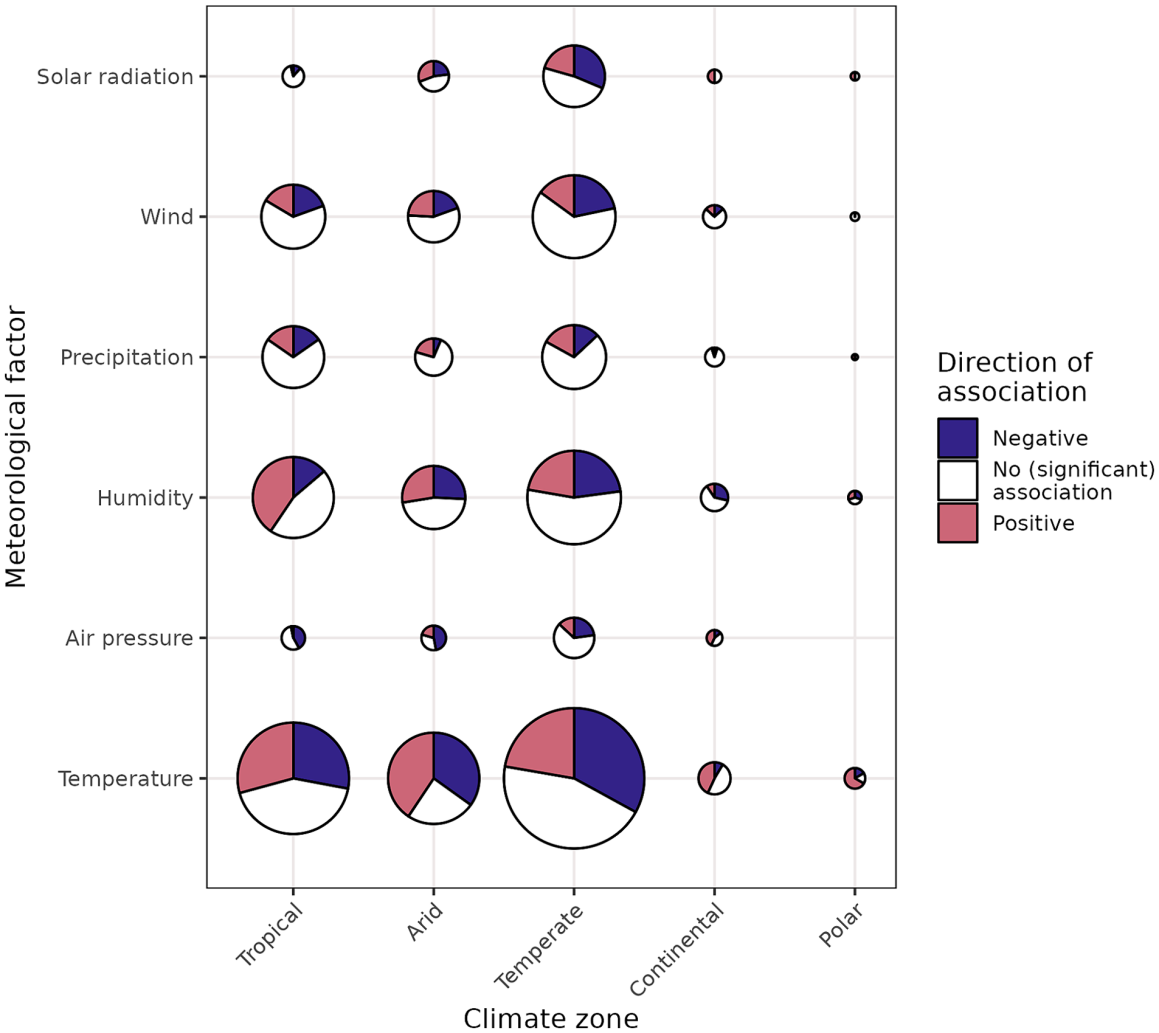
Bubble plot of the associations between meteorological factors and COVID-19 per climate zone (specific locations only). Bubble size indicates number of associations. The largest number of associations found was 1,174 (temperature for the Temperate climate zone), while the smallest number was 4 (solar radiation for the continental climate zone).

Individual associations found in the 445 other studies were grouped, both by WHO region (Figure 4) and by climate zone (Figure 5). This primarily demonstrated that the direction of the found associations was highly variable, and that many associations were not significant. Contrary to the findings of the experimental studies, non-experimental associations with temperature and solar radiation were not consistently negative, both when considering WHO regions and climate zones.

**Figure 6:**
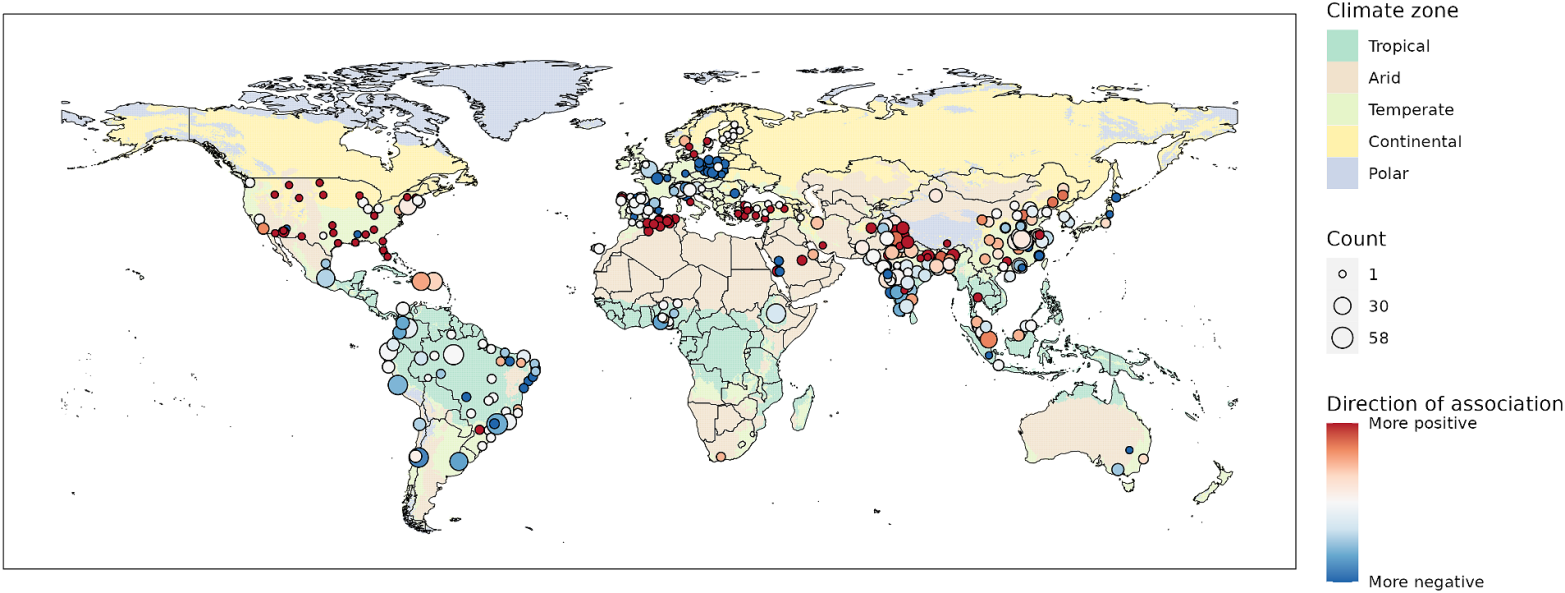
Association with temperature for specific locations. Background shows main climate zones. For visualization purposes the original climate zone grid was aggregated by a factor 2.

Associations between temperature and COVID-19 were more often positive than negative for the Eastern Mediterranean and South-East Asian regions. Associations between solar radiation and COVID-19 were more often negative than positive for all regions except the Western Pacific region. It is notable that associations between COVID-19 and all meteorological variables except for precipitation were consistently more negative than positive for the European region, and the temperate climate zone. Looking at the associations found for specific locations (as demonstrated in Figure 6 for temperature) did not reveal any obvious spatial patterns, for example with regard to latitude. For some regions, these more detailed results provide some consistent evidence at the national level. For example, the negative association between meteorological variables and COVID-19 in Europe becomes clear at this greater level of detail. For other countries, such as China and India, it reveals the large geographical variations of observed associations (Figure 6). In addition to positive and negative associations, non-significant associations were found for many specific locations, especially for precipitation and wind (Section 2 of Supplementary Data Sheet S1).

#### 3.2.3 Interactions

The extracted associations were mostly correlation coefficients. However, many studies conducted additional analyses to provide further insights. Multiple regression methods were used in several studies to describe effects in isolation. For example, one study used a generalized linear mixed model to assess associations between meteorological variables and COVID-19, while also considering potentially modifying or confounding factors such as demographics and air travel (44). An important factor to consider here is that variables might interact. Potential interaction cannot only occur between meteorological and non-meteorological factors; experimental studies demonstrated that the effects of meteorological factors on SARS-CoV-2 are not independent but may also interact, as for instance demonstrated by Dabisch and colleagues (see “Evidence from experimental and simulation studies on SARS-CoV-2”) (34). This warrants consideration of interaction effects in observational research.

**Figure 7:**
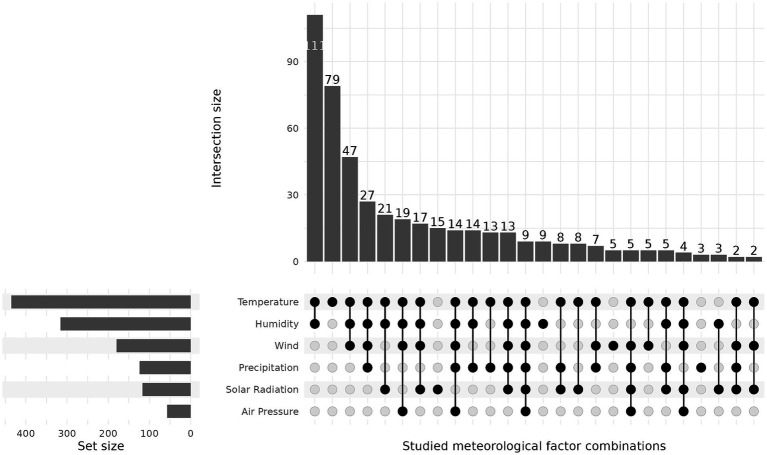
Upset plot including all studies showing combinations of meteorological factors studied. Only combinations occurring more than once are presented in this figure. The set size indicates the number of studies covering an individual meteorological factor.

Most studies examined multiple meteorological factors, with temperature and humidity being the most studied combination (Figure 7). However, these were mainly analyzed in a bivariate fashion (i.e., studies including multiple meteorological factors typically examined the association between each meteorological variable and COVID-19 separately). Nine studies investigated all meteorological factors considered in our review, but none of these studies addressed interactions.

Some observational studies analyzed the effect of interaction between multiple meteorological factors on COVID-19 outcomes. One study found that the association between relative humidity and COVID-19 was positive at low temperature (below 25°C), and negative at high temperature (above 25°C) (45), comparable to findings from Zhai and colleagues (46). Wei and colleagues incorporated multiple meteorological factors in an interaction and found a relatively higher COVID-19 risk for lower temperatures in combination with moderate precipitation (50–120 mm) (47). In the same study, increased temperatures in combination with higher wind speed were also found to be associated with increased COVID-19 transmission (47). Clouston and colleagues (48) found a significant association between low wind speed (<8.85 km/h) on days with temperatures between 16 and 28°C.

Many studies considered non-meteorological variables. Air pollution was the most frequently considered non-meteorological variable, and was shown in several studies to be both associated with COVID-19 outcomes as well as meteorological factors [e.g., (49–51)]. A study in Turkey showed that wind and population density were the factors most strongly (positively) associated with COVID-19 cases, but the effect of wind was fully mediated by population density (4). A study in the Netherlands demonstrated that inclusion of mobility (visits to indoor recreational locations) significantly improved a predictive COVID-19 model compared to a model with only environmental variables including temperature and solar radiation, indicating that weather-related changes in mobility could partially explain seasonal incidence patterns observed over longer time periods (52). Pequeno and colleagues found a negative association between temperature and COVID-19, whereas population density and number of arriving flights were positively associated with COVID-19 (44). In this study, interaction effects between time and temperature were negative, highlighting the temperature sensitivity of COVID-19 dynamics. Time and confirmed COVID-19 cases were positively correlated, meaning that cases increased with time. When including temperature in the model, higher temperature (median of 28°C compared to 21°C) weakened this effect.

## 4 Discussion

This scoping review was conducted to improve understanding of the association between meteorological factors and SARS-CoV-2/COVID-19. It presents the available knowledge on meteorological sensitivity of SARS-CoV-2 and COVID-19 epidemiology published up to January 2023, and thus includes the majority of the COVID-19 pandemic. Experimental studies provide fairly consistent evidence for direct effects of some meteorological factors on SARS-CoV-2, though it should be noted these studies were conducted in a controlled setting. Observational research showed inconsistent results both across and within regions. Our results are in line with previous studies in that it remains difficult to establish a clear relation between meteorological factors and COVID-19 from epidemiological observational studies (53). However, through our assessment we provide more in-depth guidance on how to further untangle this relation in future research. From our review, it became apparent that there is limited consideration for non-linear effects, and interactions between meteorological factors as well as demographic and environmental factors, even though these are likely to play an important role in shaping any observed association. In a real-life setting there is a constant interplay between meteorological factors. The insight offered by these studies on the associations between meteorological factors and epidemiology of COVID-19 is thus likely to be incomplete.

Although it is now well known that SARS-CoV-2 transmission primarily occurs through droplets, the majority of experimental studies analyzed the inactivation of SARS-CoV-2 by meteorological factors on surfaces. Thus, further studies on aerosolized SARS-CoV-2 under the influence of (multiple) meteorological factors would be of particular interest. For example, Feng and colleagues showed that high relative humidity enlarges droplets, which causes droplets to deposit (54). They highlighted that smaller droplets (at lower relative humidity) can remain in the air for longer periods of time and could be transported further at higher wind speed. This study did not consider that wind can also have a diluting effect, and could thus reduce the risk of exposure outdoors.

Despite analyzing different variants, the experimental studies about the effects of temperature and solar radiation on SARS-CoV-2 were consistent. This indicates that differences in observed associations between meteorological factors and COVID-19 over longer time periods were unlikely to be caused by the emergence of new virus variants (28). Viruses with different transmission pathways have been shown in previous research to be sensitive to climatic variables including temperature (55). We emphasize that the effect of temperature on viruses also applies to the matrix air.

The scoping review methodology allows for the inclusion of articles with different approaches. This makes more advanced comparison and interpretation (e.g., through meta-analysis) unfeasible. We observed that the found associations varied widely. Grouping either by WHO region or climate zone demonstrated that this heterogeneity remains when zooming into smaller, more comparable, regions. Looking at results for specific locations furthermore shows that individual studies can find different effects for the same locations. From this we can primarily conclude that the relationship between meteorological factors and COVID-19 epidemiology is complex. Tan & Schultz tried to explain these inconsistencies with a meta-regression analysis, which considered observational studies only (56). They emphasized the importance of considering the delay (lag) between infection and an examined COVID-19 outcome.

General consensus is that SARS-CoV-2 transmission primarily occurs indoors (57). Observational research included in this study typically used (outdoor) meteorological data. Thus, these studies primarily examined associations between the weather and disease acquired through indoor transmission. Weather influences the indoor environment both directly (58), and also guides how we regulate the indoor climate, for example by closing windows or turning on the heating. Additionally, weather influences how we use indoor or outdoor spaces; bad weather could move people indoors, where conditions are generally more conducive to SARS-CoV-2 transmission (52, 59). Behavioral responses to the weather might be different between cultures, which could play a part in the observed inconsistency of global findings. Future research should offer insight into these context-specific and weather-driven behavioral aspects. This could assist policymakers in developing targeted interventions (60).

Many studies acknowledged that other non-meteorological factors might also contribute to the local variation in the found associations. We found that studies did not always account for these effects. Examples of variables that were considered include lockdown policies, air pollution, population density, mobility, or health status. Some of these variables can interact with or modify effects of meteorological variables. A study by Zhang and colleagues, for example, examined the interaction between temperature and air pollution and found a moderating effect of air pollution on the relationship between temperature and COVID-19 (61). Overall, a negative association between temperature and COVID-19 was found. However, in northern China, characterized by relatively poor air quality during the study period, air pollution was found to ‘reverse’ the direction of the effect of temperature on COVID-19. Here, increasing temperatures strengthened the effects of air pollution on COVID-19 (61). Other variables might act as confounders. For example, strict lockdown measures enacted during a period of cooling weather might lead to the impression of a positive association between temperature and COVID-19.

Compared to similar pathogens and infectious diseases, the body of research on the relationship between meteorological variables and SARS-CoV-2/COVID-19 is substantial. About 4% of the total scientific research output in 2020 was devoted to SARS-CoV-2/COVID-19 (62), and research output relevant to our review remained high throughout the study period. This unprecedented volume of published papers could have led to added pressure on academics to quickly publish findings and on editors to release potentially important information for healthcare practitioners and policy makers (62, 63). Combined with the fact that COVID-19 was a new disease, this may have contributed to methodological shortcomings, even in peer-reviewed research. We did not make a formal assessment of study quality but did make note of several potential quality issues, such as missing significance levels or data sources. An important general observation is that the associations identified in this review primarily cover 2020, despite including studies published up to January 2023. As such, most study periods were characterized by highly unusual epidemic dynamics, and this may have influenced study results. Nonetheless, inclusion of studies covering longer time periods did not reveal novel insights compared to research conducted earlier in the pandemic.

An important gap to address would be the consensus in methodology between studies. An approach where researchers agree and collaborate on a comparable approach in terms of (non-)meteorological factors studied, temporal or spatial resolution and statistical methods would be key to increase reproducibility and allow collective interpretation of the weather - COVID-19 associations. Additionally, large differences in availability of data between regions will have introduced bias in our results. We found little evidence for the African and Eastern Mediterranean regions, and more specifically for Eastern Europe, Central Asia, and the South-East Asian region outside of India. Differences could be explained (in part) by differences in access to testing, testing behavior, and testing policies. But also more generally by differences in reporting of data, and access to research funding. Our exclusion of non-scientific literature and studies not published in English has also contributed to this bias. Associations between meteorological factors and COVID-19 show strong local variation. As such, future research should focus on addressing the limited evidence for certain world regions.

Seasonality is often observed for viral (respiratory) infectious diseases, and studies on human coronaviruses present an opportunity to close knowledge gaps in underlying connections between virus dynamics and weather. Other respiratory infectious diseases such as influenza and diseases caused by other human coronaviruses show seasonal patterns as well (1, 2, 5). SARS-CoV, MERS and other respiratory viruses with pandemic potential were previously shown to be climate sensitive. Consequently, climate change could have potentially far-reaching impacts on infectious diseases. Considering this characteristic may thus be important in the context of pandemic preparedness. Insights from this study about impacts of meteorological factors on COVID-19 dynamics can improve epidemiological predictability. Our findings also highlight the complexity introduced by (local-level) effects of interactions and non-meteorological variables, which should be considered in outbreak modeling. Since meteorological factors vary within countries, it is recommended that pandemic preparedness policies should consider the importance of the local context and accommodate a more targeted response. It is necessary to further study the climate-sensitive aspects of (emerging) pathogens to predict effects of health and diseases in changing climates (64, 65).

It is clear that meteorological factors play a role in COVID-19 dynamics. This effect appears to be complex and shaped by both direct and indirect processes. Experimental studies about SARS-CoV-2, providing evidence for direct effects on the virus but not necessarily for the overall transmission process, showed that higher temperatures and solar radiation decrease virus viability. For the remaining meteorological factors, such as humidity, air pressure, precipitation, and wind, no consistent evidence was found. Observational studies which had COVID-19 as an outcome did not always align with the expectations set by experimental studies. Even after clustering the results from observational studies by WHO regions and climate zones, no consistent patterns could be identified. Examining results for specific locations also demonstrated high variability within countries. Our findings imply that local variation in (interactions between) meteorological factors and non-meteorological factors (e.g., behavior) are essential to consider when studying how meteorological factors affect COVID-19 dynamics. This is supported by studies showing that factors such as population density, mobility or air pollution can influence the association between meteorological factors and COVID-19. Although weather seems to play an indirect role in the transmission of COVID-19, experimental and observational studies indicate that increased temperature and solar radiation impair virus survival and possibly transmission. Future research could close research gaps through a more harmonized research approach, substantiated by evidence from experimental studies. This would help to reach consensus on the influence of meteorological factors on human coronaviruses and other respiratory viruses with pandemic potential.

## Data availability statement

The original contributions presented in the study are included in the article/Supplementary material, further inquiries can be directed to the corresponding author.

## Author contributions

JL and AR conceptualized the study and acquired the research funding. JL was responsible for project management. JL, SF, and LB designed the review protocol. SF and LB collected the literature. JL, AL, SF, and LB reviewed the titles and abstracts. SF, LB, JL, SD, AL, and LV reviewed the full texts and extracted the data. JL, SD, SF, and LB curated the data and wrote the original draft. JL and SD made data visualizations. JL, SD, AR, LV, and AL reviewed and edited the manuscript. All authors contributed to the article and approved the submitted version.
