# Supplementary material for: Associations between meteorological factors and COVID-19: a global scoping review
Source: Front Public Health. 2024 Jul 18;12:1183706. doi: 10.3389/fpubh.2024.1183706 (PMC11291467; doi:10.3389/fpubh.2024.1183706)
Supplement: Supplementary file 1 [file Data_Sheet_1.docx]

Supplementary Material

Associations between meteorological factors and COVID-19: a global scoping review

Jesse Limaheluw^1, 2, † *^, Sophia Dollmann^1, 2, †^, Sofia Folpmers^1, 2^, Lola Beltrán Beut^1,2^, Afroditi Lazarakou^1^, Lucie C. Vermeulen^1^, Ana Maria de Roda Husman^1, 2^

*** Correspondence:** Corresponding Author: Jesse Limaheluw, jesse.limaheluw@rivm.nl

# Full search queries per database

## Embase

('SARS-related coronavirus'/exp OR 'SARS-CoV*':ti,ab OR 'severe acute respiratory syndrome coronavirus*':ti,ab OR 'novel coronavirus':ti,ab OR 'new coronavirus':ti,ab OR 'Coronavirus infection':ti,ab OR 'coronavirus disease 2019':ti,ab OR 'Covid-19':ti,ab OR 'severe acute respiratory syndrome'/exp OR 'severe acute respiratory syndrome':ti,ab OR 'SARS':ti,ab OR 'Middle East respiratory syndrome'/exp OR 'Middle East respiratory syndrome':ti,ab OR 'MERS':ti,ab OR 'Middle East respiratory syndrome coronavirus'/exp OR 'Middle East respiratory syndrome coronavirus':ti,ab OR 'MERS-CoV':ti,ab OR 'Human coronavirus NL63'/exp OR ‘Human coronavirus NL63’:ti,ab OR 'HCoV-NL63':ti,ab OR 'Human coronavirus OC43/exp' OR ‘Human coronavirus OC43’:ti,ab OR 'HCoV-OC43':ti,ab OR 'Human coronavirus 229E'/exp OR ‘Human coronavirus 229E’:ti,ab OR 'HCoV-229E':ti,ab OR 'human coronavirus hku1'/exp OR 'human coronavirus HKU1':ti,ab OR 'HCoV-HKU1':ti,ab OR 'Human coronavir*':ti,ab OR 'HCoV':ti,ab)

AND

('climate'/exp OR 'climate':ti,ab OR 'climat* condition*':ti,ab OR 'climat* factor*':ti,ab NOT ('climate change'/exp OR 'climate change':ti,ab,kw) OR 'meteorological condition*':ti,ab OR 'meteorological factor*':ti,ab OR 'weather'/exp OR 'weather':ti,ab OR 'rain':ti,ab OR 'rainfall':ti,ab OR 'precipitation'/exp OR 'precipitation':ti,ab OR 'wind':ti,ab OR 'cloud*':ti,ab OR 'humidity'/exp OR 'humidity':ti,ab OR 'solar radiation'/exp OR 'solar radiation':ti,ab OR 'sunlight'/exp OR 'sunlight':ti,ab OR 'sunshine':ti,ab OR 'air temperature'/exp OR 'air temperature':ti,ab OR 'environmental temperature'/exp OR 'environmental temperature':ti,ab OR 'ambient temperature':ti,ab OR 'atmospheric pressure'/exp OR 'atmospheric pressure':ti,ab OR 'air pressure':ti,ab)

## PubMed

("SARS Virus" [MH] OR “SARS-related coronavirus”[TIAB] OR “SARS-CoV*”[TIAB] OR “severe acute respiratory syndrome coronavirus”[TIAB] OR “novel coronavirus”[TIAB] OR “new coronavirus”[TIAB] OR “Coronavirus infection”[TIAB] OR “coronavirus disease 2019”[TIAB] OR “Covid-19”[TIAB] OR “severe acute respiratory syndrome”[MH] OR “severe acute respiratory syndrome”[TIAB] OR “SARS”[TIAB] OR “Middle East respiratory syndrome”[TIAB] OR “MERS”[TIAB] OR “Middle East respiratory syndrome coronavirus”[MH] OR “Middle East respiratory syndrome coronavirus”[TIAB] OR “MERS-CoV”[TIAB] OR "Coronavirus NL63, Human" [MH] OR “Human coronavirus NL63”[TIAB] OR “HCoV-NL63”[TIAB] OR "Coronavirus OC43, Human" [MH] OR “Human coronavirus OC43”[TIAB] OR “HCoV-OC43”[TIAB] OR "Coronavirus 229E, Human" [MH] OR “Human coronavirus 229E”[TIAB] OR “HCoV-229E”[TIAB] OR “human coronavirus HKU1”[TIAB] OR “HCoV-HKU1”[TIAB] OR “Human coronavir*”[TIAB] OR “HCoV”[TIAB])

AND

(“climate”[MH] OR “climate”[TIAB] OR “climatic condition*”[TIAB] OR “climatic factor*”[TIAB] NOT (“climate change”[MH] OR “climate change”[TIAB]) OR “meteorological condition*”[TIAB] OR “meteorological factor*”[TIAB] OR “weather”[MH] OR “weather”[TIAB] OR “rain”[TIAB] OR “rainfall”[TIAB] OR “precipitation”[TIAB] OR “wind”[TIAB] OR “cloud*”[TIAB] OR “humidity”[MH] OR “humidity”[TIAB] OR “solar radiation”[TIAB] OR “sunlight”[MH] OR “sunlight”[TIAB] OR “sunshine”[TIAB] OR "temperature" [MH] OR “air temperature”[TIAB] OR “environmental temperature”[TIAB] OR “ambient temperature”[TIAB] OR “atmospheric pressure”[MH] OR “atmospheric pressure”[TIAB] OR “air pressure”[TIAB])

## Scopus

( TITLE-ABS ( "SARS-related coronavirus" ) OR TITLE-ABS ( "SARS-CoV*" ) OR TITLE-ABS ( "severe acute respiratory syndrome coronavirus*" ) OR TITLE-ABS ( "novel coronavirus" ) OR TITLE-ABS ( "new coronavirus" ) OR TITLE-ABS ( "Coronavirus infection" ) OR TITLE-ABS ( "coronavirus disease 2019" ) OR TITLE-ABS ( "Covid-19" ) OR TITLE-ABS ( "severe acute respiratory syndrome" ) OR TITLE-ABS ( "SARS" ) OR TITLE-ABS ( "Middle East respiratory syndrome" ) OR TITLE-ABS ( "MERS" ) OR ( "Middle East respiratory syndrome coronavirus" ) OR TITLE-ABS ( "MERS-CoV" ) OR TITLE-ABS ( "Human coronavirus NL63" ) OR TITLE-ABS ( "HCoV-NL63" ) OR TITLE-ABS ( "Human coronavirus OC43" ) OR TITLE-ABS ( "HCoV-OC43" ) OR TITLE-ABS ( "Human coronavirus 229E" ) OR TITLE-ABS ( "HCoV-229E" ) OR TITLE-ABS ( "human coronavirus HKU1" ) OR TITLE-ABS ( "HCoV-HKU1" ) OR TITLE-ABS ( "Human coronavir*" ) OR TITLE-ABS ( "HCoV" ) )

AND

( TITLE-ABS ( "climate" ) OR TITLE-ABS ( "climat* condition*" ) OR TITLE-ABS ( "climat* factor*" ) AND NOT ( TITLE-ABS ( "climate change" ) ) OR TITLE-ABS ( "meteorological condition*" ) OR TITLE-ABS ( "meteorological factor*" ) OR TITLE-ABS ( "weather" ) OR TITLE-ABS ( "rain" ) OR TITLE-ABS ( "rainfall" ) OR TITLE-ABS ( "precipitation" ) OR TITLE-ABS ( "wind" ) OR TITLE-ABS ( "cloud*" ) OR TITLE-ABS ( "humidity" ) OR TITLE-ABS ( "solar radiation" ) OR TITLE-ABS ( "sunlight" ) OR TITLE-ABS ( "sunshine" ) OR TITLE-ABS ( "air temperature" ) OR TITLE-ABS ( "environmental temperature" ) OR TITLE-ABS ( "ambient temperature" ) OR TITLE-ABS ( "atmospheric pressure" ) OR TITLE-ABS ( "air pressure" ) )

# Additional world maps showing associations between specific meteorological factors and COVID-19

***
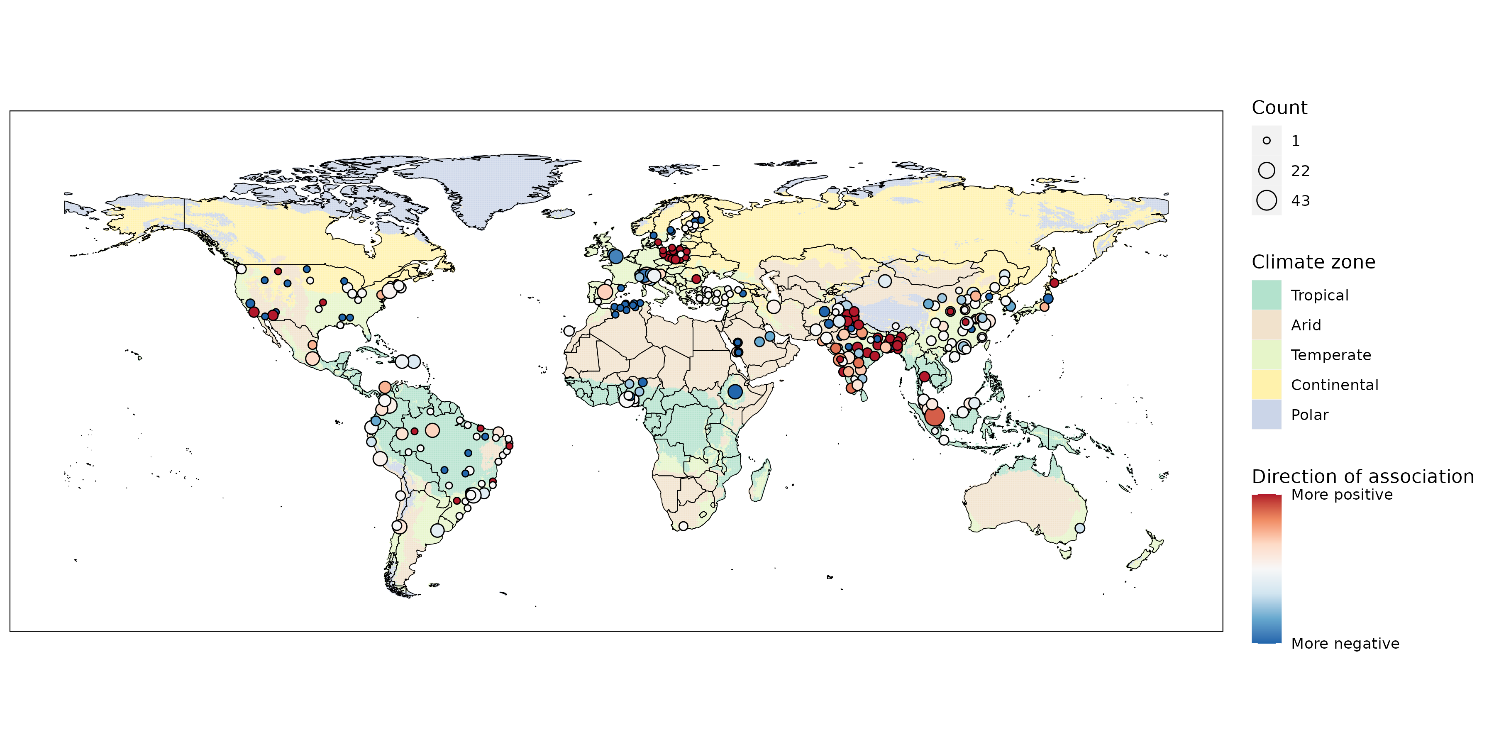
***

**Figure S1** Association with humidity for specific locations. Background shows main climate zones.


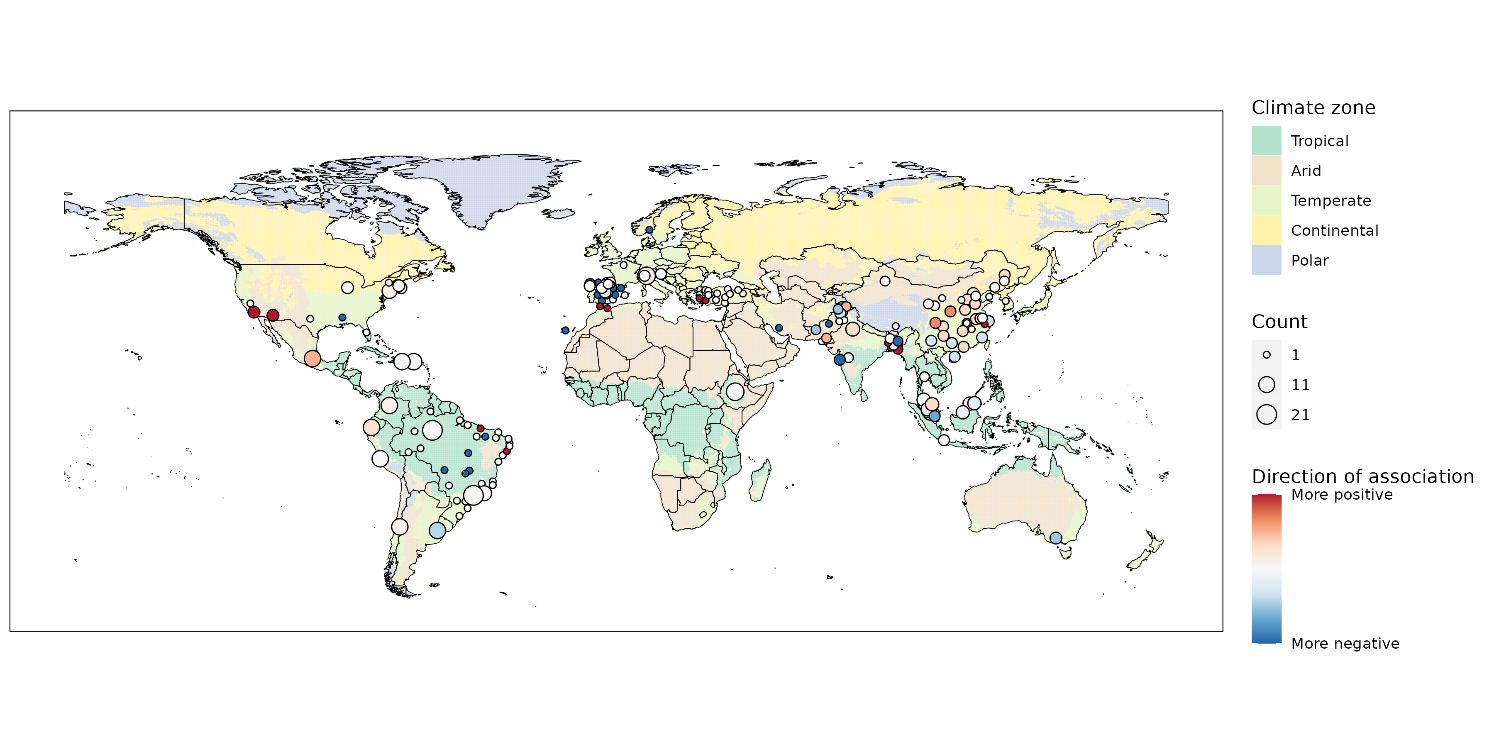


**Figure S2** Association with precipitation for specific locations. Background shows main climate zones.


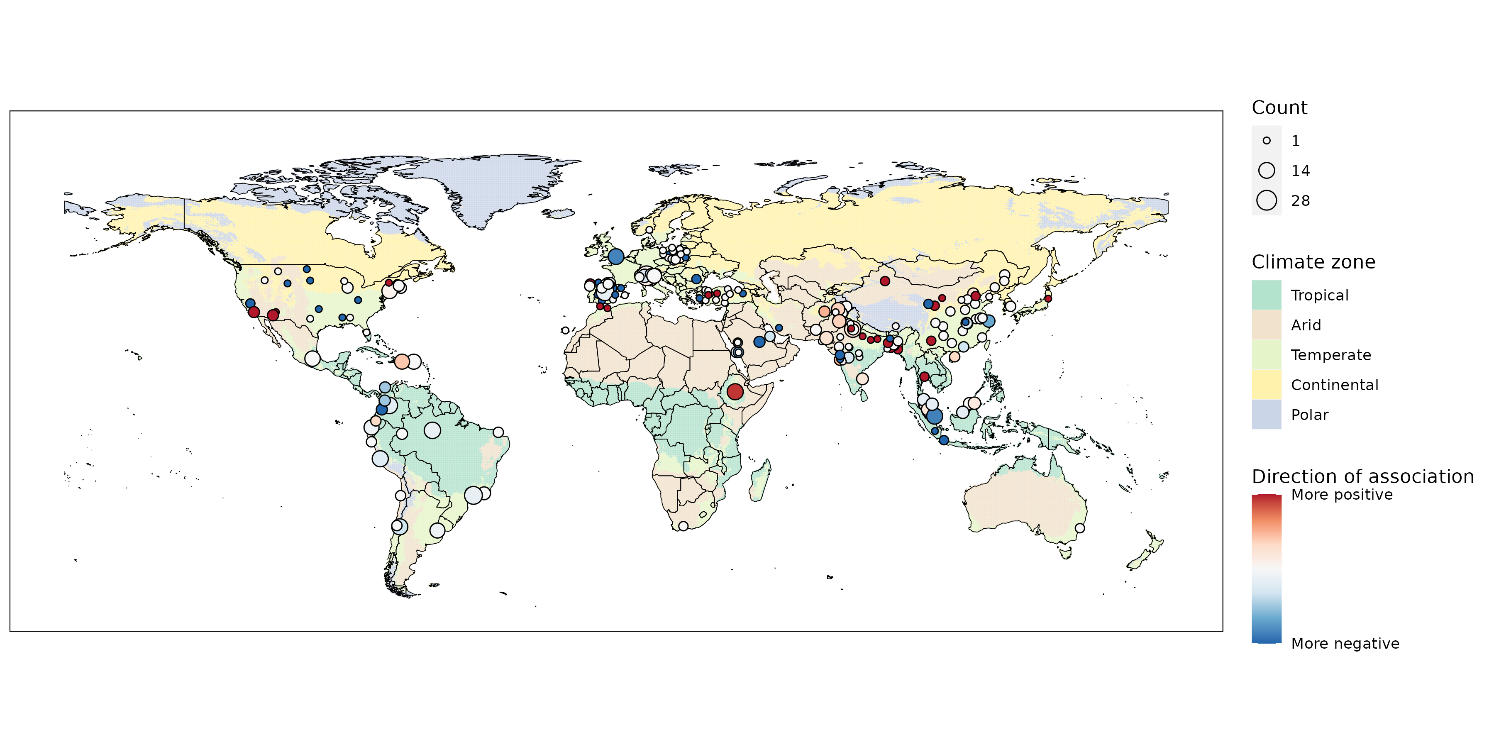


**Figure S3** Association with wind for specific locations. Background shows main climate zones.


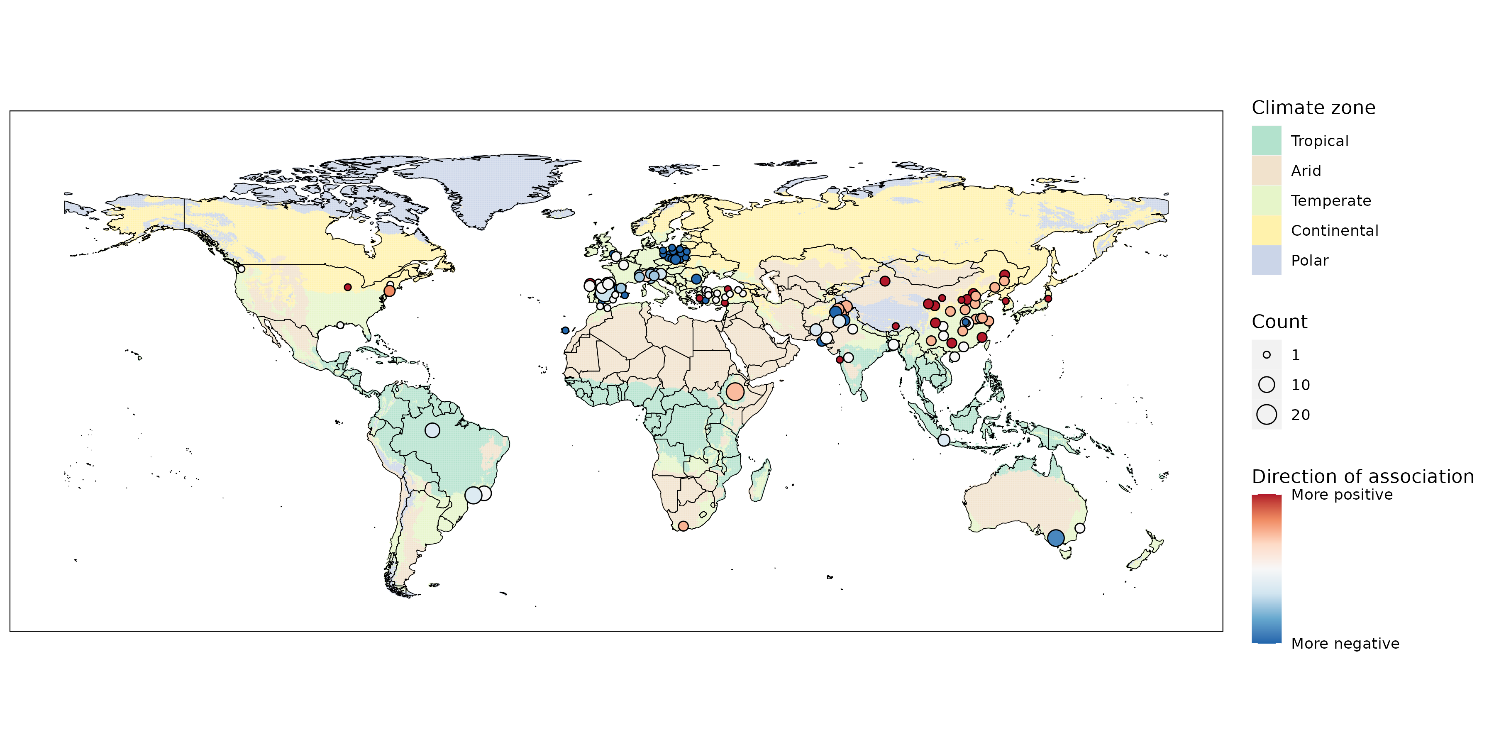


**Figure S4** Association with solar radiation for specific locations. Background shows main climate zones.


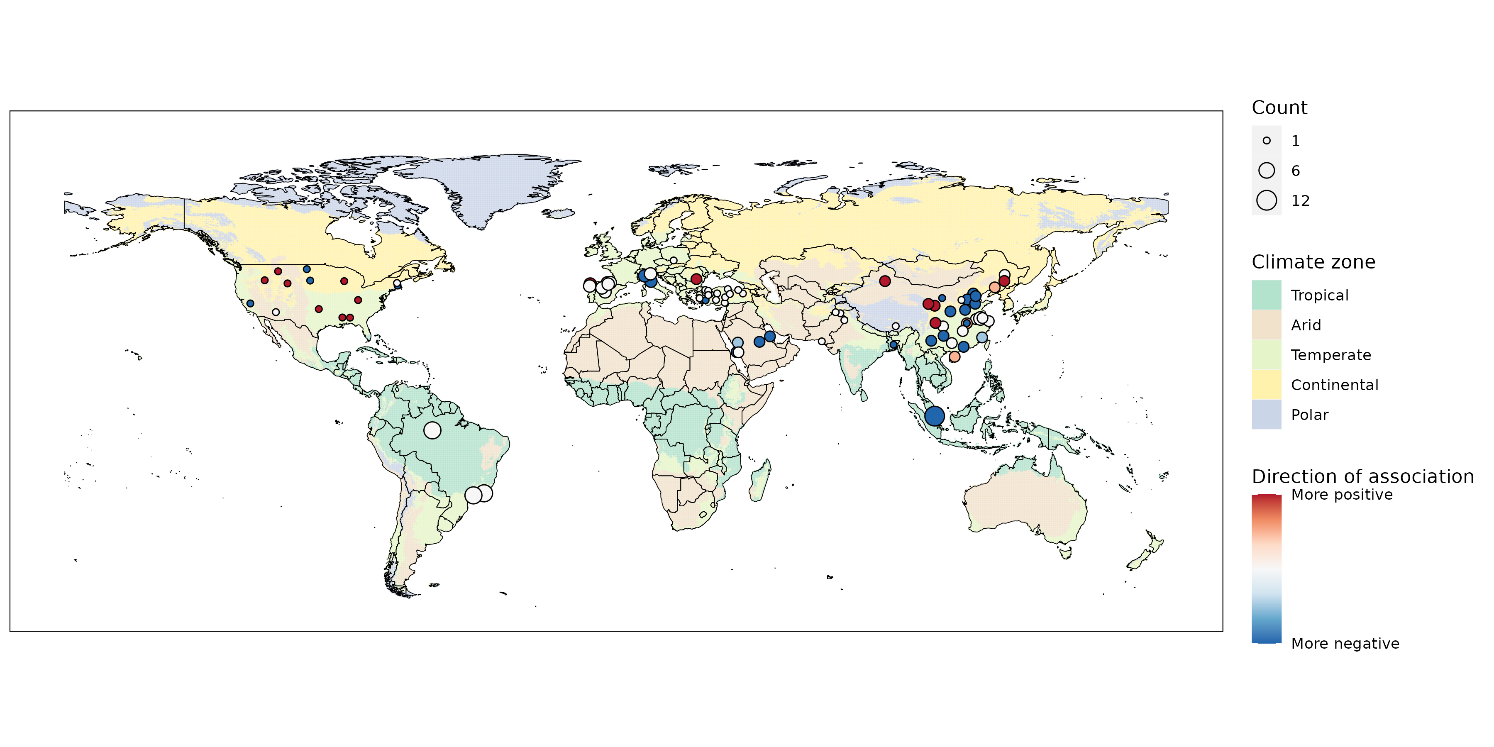


**Figure S5** Association with air pressure for specific locations. Background shows main climate zones.
